# Nonlinear Generation of Fluting Perturbations by Kink Mode

**DOI:** 10.1007/s11207-017-1133-0

**Published:** 2017-08-11

**Authors:** M. S. Ruderman

**Affiliations:** 1 Solar Physics and Space Plasma Research Centre (SP²RC), School of Mathematics and Statistics, University of Sheffield, Hicks Building, Hounsfield Road, Sheffield, S3 7RH UK; 2 Space Research Institute (IKI), Russian Academy of Sciences, Moscow, Russia

**Keywords:** Sun, corona, Magnetic fields, Magnetohydrodynamics, Waves, Oscillations

## Abstract

We study the excitation of fluting perturbations in a magnetic tube by an initially imposed kink mode. We use the ideal magnetohydrodynamic (MHD) equations in the cold-plasma approximation. We also use the thin-tube approximation and scale the dependent and independent variables accordingly. Then we assume that the dimensionless amplitude of the kink mode is small and use it as an expansion parameter in the regular perturbation method. We obtain the expression for the tube boundary perturbation in the second-order approximation. This perturbation is a superposition of sausage and fluting perturbations. The amplitude of the fluting perturbation takes its maximum at the middle of the tube, and it monotonically decreases with the distance from the middle of the tube.

## Introduction

After transverse oscillations of coronal magnetic loops were first observed by the *Transition Region and Coronal Explorer* (TRACE) in 1998 and were reported by Aschwanden *et al.* (1999) and Nakariakov *et al.* (1999), they attracted enhanced attention from theorists. These oscillations were interpreted as fast kink standing waves in magnetic flux tubes. Initially the simplest model of a straight homogeneous magnetic tube (*e.g.* Ryutov and Ryutova, 1976; Edwin and Roberts, 1983) was used for the theoretical studies of coronal-loop transverse oscillations. Later more sophisticated models taking into account such effects as the plasma density variation along and across a tube, the presence of flows, and loop cooling were developed. For a review of the theory of coronal-loop oscillations see, *e.g.*, Ruderman and Erdélyi (2009).

The majority of studies on coronal-loop kink oscillations were carried out in the approximation of linear magnetohydrodynamics (MHD). Studies of nonlinear coronal-loop kink oscillations are sparse. Ruderman (1992) and Ruderman, Goossens, and Andries (2010) analytically studied nonlinear propagating kink waves. Ruderman and Goossens (2014) also analytically investigated the effect of nonlinearity on standing kink waves in magnetic tubes with the density varying along the tube. There were also a few numerical studies of nonlinear kink oscillations of magnetic tubes (*e.g.* Terradas *et al.*, 2008; Magyar, Van Doorselaere, and Marcu, 2015; Magyar and Van Doorselaere, 2016).

This study is motivated by the discussion in a meeting of an international group led by G. Verth and R. Morton at the International Space Science Institute (ISSI). In this meeting (Terradas, Magyar, and Van Doorsselaere 2017) presented the results of the study of nonlinear kink oscillations of a magnetic tube. In particular, they reported the appearance of a fluting perturbation of the tube boundary excited by an initially imposed kink oscillation. The period of the fluting perturbation was equal to the half period of the kink oscillation, and its amplitude took its maximum at the centre of the magnetic tube. Some of the meeting participants insisted that the fluting perturbation must be the first harmonic of the first fluting mode. The amplitude of this harmonic is zero at the tube centre.

Ruderman, Goossens, and Andries (2010) and Ruderman and Goossens (2014) predicted the excitation of the fluting perturbation with the frequency equal to the double frequency of the kink mode. They also predicted that the amplitude of the fluting perturbation is proportional to the amplitude of the kink mode squared. However, their results cannot be directly compared with those reported by Terradas, Magyar, and Van Doorsselaere (2017) because Ruderman, Goossens, and Andries (2010) studied propagating waves, and Ruderman and Goossens (2014) concentrated on the nonlinearity effect on the kink oscillations of a magnetic tube strongly stratified in the longitudinal direction.

This article aims to study analytically the excitation of a fluting perturbation by an initially imposed kink mode and compare the analytical results with the numerical results obtained by Terradas, Magyar, and Van Doorsselaere (2017). The article is organised as follows. In the next section we formulate the problem and write down the governing equations and boundary conditions. In Section 3 we use the regular perturbation method to study the excitation of fluting perturbation. Section 4 contains the summary of the results obtained and our conclusions.

## Problem Formulation and Governing Equations

We use the cold-plasma and thin-tube approximations. In the unperturbed state $\boldsymbol{B} = B\boldsymbol{e}_{z}$, where $\boldsymbol{e}_{z}$ is the unit vector in the $z$-direction of cylindrical coordinates $r$, $\phi$, $z$. The equilibrium density $[\rho]$ is given by

1 $$ \rho = \left \{\textstyle\begin{array}{l@{\quad}l} \rho_{i}, & r < R, \\ \rho_{e}, & r > R,\end{array}\displaystyle \right . $$

where $\rho_{i}$ and $\rho_{e}$ are constant, and $\rho_{e} < \rho_{i}$. The perturbations are governed by the ideal MHD equations,

2 $$\begin{aligned} \rho \biggl(\frac{\partial\boldsymbol{v}}{\partial t} + (\boldsymbol{v}\cdot\nabla)\boldsymbol{v} \biggr) &= \frac{1}{\mu_{0}}(\nabla\times\boldsymbol{b})\times (\boldsymbol{B} + \boldsymbol{b}) , \end{aligned}$$

3 $$\begin{aligned} \ \frac{\partial\boldsymbol{b}}{\partial t} &= \nabla\times\bigl[\boldsymbol{v}\times (\boldsymbol{B} + \boldsymbol{b})\bigr], \end{aligned}$$

where $\rho$ is the density, $\boldsymbol{v}$ the plasma velocity, $\boldsymbol{b}$ the magnetic-field perturbation, and $\mu_{0}$ the magnetic permeability of free space. The perturbations must satisfy the frozen-in conditions at the tube footpoints,

4 $$ \boldsymbol{v}_{\perp}=0 \quad \mbox{at} \ z = \pm L/2, $$

where $L$ is the tube length and $\boldsymbol{v}_{\perp}= \boldsymbol{v} - \boldsymbol{e}_{z} (\boldsymbol{v}\cdot\boldsymbol{e}_{z})$. We also impose the boundary conditions at the tube boundary,

5 $$ \boldsymbol{v}\cdot\boldsymbol{e}_{r} = \frac{\partial\eta}{\partial t} + \boldsymbol{v}\cdot\nabla\eta , \qquad \bigl[\!\!\bigl[2\boldsymbol{b}\cdot\boldsymbol{B} + |\boldsymbol{b}|^{2} \bigr]\!\!\bigr]= 0 \quad \mbox{at} \ r = R + \eta(t, \phi,z), $$

where $\eta(t,\phi,z)$ is the tube boundary perturbation, $\boldsymbol{e}_{r}$ is the unit vector in the radial direction, and the double brackets indicate the jump of a quantity across the tube boundary. For an arbitrary function $[f]$ this jump is defined as

$$[\!\![f ]\!\!]= \lim_{\varepsilon \to +0}\bigl[f(R + \eta + \varepsilon) - f(R + \eta - \varepsilon)\bigr]. $$

Below we assume that the tube is thin: $R/L = \epsilon \ll 1$. In accordance with this assumption we introduce the stretching variable $Z = \epsilon z$. The characteristic alfvénic time related to the tube radius is $R/V_{A}$, where $V_{A} = B(\mu_{0}\rho)^{-1/2}$ is the Alfvén speed. It can be the Alfvén speed either inside or outside the tube because we assume that the density ratio $[\rho_{i}/\rho_{e}]$ is not large, implying that the two Alfvén speeds are of the same order. On the other hand, the oscillation period is of the order of $L/V_{A} = \epsilon^{-1}R/V_{A}$. This inspires us to introduce the “slow” time $T = \epsilon t$. Below we assume that the maximum tube axis displacement is of the order of $aR$, where $a \ll 1$. The quantity $a$ can be considered as the dimensionless amplitude of the tube kink oscillation. Later we shall assume that, although $a$ is small, $a \gg \epsilon$.

In accordance with the definition, $\eta/R = \mathcal{O}(a)$. We now obtain similar estimates for other variables. Although we use nonlinear equations, the nonlinear correction to the linear solution will be small. Then we can use the estimates for the order of magnitude of perturbation of various quantities obtained using the linear theory. Then it follows that the ratios of the radial and azimuthal components of the velocity to $V_{A}$ is of the order of $\epsilon a$, and the same is true for the ratios of the radial and azimuthal components of the magnetic-field perturbation to $B$. On the other hand, the ratio of the $z$-component of the velocity to $V_{A}$, and the ratio of the $z$-component of the magnetic field to $B$ are both of the order of $\epsilon^{2} a$. In accordance with these estimates we introduce the scaled components of the velocity and magnetic-field perturbation and write

6 $$ \boldsymbol{v} = \bigl(\epsilon v_{r},\epsilon v_{\phi}, \epsilon^{2} v_{z}\bigr), \qquad \boldsymbol{b} = \bigl( \epsilon b_{r},\epsilon b_{\phi},\epsilon^{2} b_{z}\bigr). $$

We now substitute these expressions in Equations 2 and 3 and write the equations obtained in components keeping only the leading terms with respect to $\epsilon$. As a result we obtain

7 $$\begin{gathered} \frac{\partial v_{r}}{\partial T} + v_{r}\frac{\partial v_{r}}{\partial r} + \frac{v_{\phi}}{r} \frac{\partial v_{r}}{\partial\phi} - \frac{v_{\phi}^{2}}{r} = -\frac{1}{\rho}\frac{\partial P}{\partial r} + \frac{1}{\mu_{0}\rho} \biggl(B\frac{\partial b_{r}}{\partial Z} + b_{r} \frac{\partial b_{r}}{\partial r} + \frac{b_{\phi}}{r}\frac{\partial b_{r}}{\partial\phi} - \frac{b_{\phi}^{2}}{r} \biggr) , \end{gathered}$$

8 $$\begin{gathered} \frac{\partial v_{\phi}}{\partial T} + v_{r}\frac{\partial v_{\phi}}{\partial r} + \frac{v_{\phi}}{r}\frac{\partial v_{\phi}}{\partial\phi} + \frac{v_{r} v_{\phi}}{r} = -\frac{1}{\rho r}\frac{\partial P}{\partial\phi} + \frac{1}{\mu_{0}\rho} \biggl(B \frac{\partial b_{\phi}}{\partial Z} + b_{r}\frac{\partial b_{\phi}}{\partial r} + \frac{b_{\phi}}{r} \frac{\partial b_{\phi}}{\partial\phi} + \frac{b_{r} b_{\phi}}{r} \biggr) , \end{gathered}$$

9 $$\begin{gathered} \frac{\partial b_{r}}{\partial T} = B\frac{\partial v_{r}}{\partial Z} + \frac{1}{r} \frac{\partial}{\partial\phi}(v_{r} b_{\phi}- v_{\phi}b_{r}), \end{gathered}$$

10 $$\begin{gathered} \frac{\partial b_{\phi}}{\partial T} = B\frac{\partial v_{\phi}}{\partial Z} - \frac{\partial}{\partial r}(v_{r} b_{\phi}- v_{\phi}b_{r}), \end{gathered}$$

11 $$\begin{gathered} \frac{\partial(rv_{r})}{\partial r} + \frac{\partial v_{\phi}}{\partial\phi} = 0, \end{gathered}$$

where

12 $$ P = \frac{1}{2\mu_{0}} \bigl(2Bb_{z} + b_{r}^{2} + b_{\phi}^{2} \bigr) $$

is the scaled perturbation of the total pressure. The boundary conditions take the form

13 $$\begin{gathered} v_{r} = v_{\phi}= 0 \quad \mbox{at} \ Z = \pm R/2 , \end{gathered}$$

14 $$\begin{gathered} v_{r} = \frac{\partial\eta}{\partial T} + \frac{v_{\phi}}{r}\frac{\partial\eta}{\partial\phi}, \qquad [\!\![P ]\!\!]= 0 \quad \mbox{at} \ r = R + \eta, \end{gathered}$$

where we took into account that $\epsilon L = R$.

An important property of this system of equations is that it does not contain $v_{z}$. What is also worth nothing is that the $z$-component of the induction equation reduces to Equation 11 which shows that the motion is incompressible in the leading-order approximation with respect to $\epsilon$.

The system of Equations 7 – 11 with the boundary conditions in Equations 13 and 14 is used in the next section to study the generation of fluting perturbations by a kink mode.

## Generation of Fluting Perturbations

We use the regular perturbation method and look for the solution to the system of Equations 7 – 11 in the form of expansions with respect to the small dimensionless wave amplitude $a$. We use the power series expansion

15 $$ f = a f_{1} + a^{2} f_{2} + \dots, $$

where $f$ is any of the dependent variables. We substitute the expansions of all dependent variables in Equations 7 – 11 and the boundary conditions in Equations 13 and 14, and collect the terms of the same order with respect to $a$.

### The First Order Approximation

In the first-order approximation we collect the terms of the order of $a$. As a result we obtain

16 $$\begin{gathered} \frac{\partial v_{r1}}{\partial T} = \frac{B}{\mu_{0}\rho}\frac{\partial b_{r1}}{\partial Z} - \frac{1}{\rho}\frac{\partial P_{1}}{\partial r}, \qquad \frac{\partial v_{\phi1}}{\partial T} = \frac{B}{\mu_{0}\rho} \frac{\partial b_{\phi1}}{\partial Z} - \frac{1}{\rho r}\frac{\partial P_{1}}{\partial\phi}, \end{gathered}$$

17 $$\begin{gathered} \frac{\partial b_{r1}}{\partial T} = B\frac{\partial v_{r1}}{\partial Z}, \qquad \frac{\partial b_{\phi1}}{\partial T} = B \frac{\partial v_{\phi1}}{\partial Z}, \qquad \frac{\partial(rv_{r1})}{\partial r} + \frac{\partial v_{\phi1}}{\partial\phi} = 0. \end{gathered}$$

In this order approximation the boundary conditions in Equations 13 and 14 reduce to

18 $$\begin{gathered} v_{r1} = v_{\phi1} = 0 \quad \mbox{at} \ Z = \pm R/2 , \end{gathered}$$

19 $$\begin{gathered} v_{r1} = \frac{\partial\eta_{1}}{\partial T}, \qquad [\!\![P_{1} ]\!\!]= 0 \quad \mbox{at}\ r = R . \end{gathered}$$

Eliminating the magnetic-field perturbation we transform Equation 16 to

20 $$\begin{aligned} \frac{\partial^{2} v_{r1}}{\partial T^{2}} - V_{A}^{2}\frac{\partial^{2} v_{r1}}{\partial Z^{2}} &= - \frac{1}{\rho}\frac{\partial^{2} P_{1}}{\partial r\partial T}, \end{aligned}$$

21 $$\begin{aligned} \frac{\partial^{2} v_{\phi1}}{\partial T^{2}} - V_{A}^{2}\frac{\partial^{2} v_{\phi1}}{\partial Z^{2}} &= - \frac{1}{\rho r}\frac{\partial^{2} P_{1}}{\partial\phi\partial T}. \end{aligned}$$

Then we use the last equation in Equation 17 to eliminate $v_{r1}$ and $v_{\phi1}$ from Equations 20 and 21. As a result, we obtain the equation for $P_{1}$:

22 $$ \frac{\partial}{\partial T} \biggl(r\frac{\partial}{\partial r}r\frac{\partial P_{1}}{\partial r} + \frac{\partial^{2} P_{1}}{\partial\phi^{2}} \biggr) = 0. $$

Imposing the condition that the solution to this equation is periodic in time with zero average over the period we reduce this equation to

23 $$ r\frac{\partial}{\partial r}r\frac{\partial P_{1}}{\partial r} + \frac{\partial^{2} P_{1}}{\partial\phi^{2}} = 0. $$

We look for the solution to this equation describing the fundamental kink mode and take $P_{1}$ proportional to $\cos\phi$. Then the solution to this equation satisfying the second boundary condition in Equation 19, regular at $r = 0$ and decaying as $r \to \infty$, is given by

24 $$ P_{1} = \widetilde{P}_{1}(T,Z) (r/R)f(r)\cos\phi, $$

where

25 $$ f(r) = \left \{\textstyle\begin{array}{l@{\quad}l} 1, & r < R,\\ (R/r)^{2}, & r > R,\end{array}\displaystyle \right . $$

and, at present, $\widetilde{P}_{1}(T,Z)$ is an arbitrary function. Writing Equation 20 at the two sides of the tube boundary and using Equation 24 and the first boundary condition in Equation 19 we obtain the system of equations for $\eta_{1}$ and $\widetilde{P}_{1}$,

26 $$\begin{aligned} \rho_{i} \biggl(\frac{\partial^{3}\eta_{1}}{\partial T^{3}} - V_{Ai}^{2} \frac{\partial^{3}\eta_{1}}{\partial T\partial Z^{2}} \biggr) &= -\frac{1}{R}\frac{\partial\widetilde{P}_{1}}{\partial T}\cos\phi, \end{aligned}$$

27 $$\begin{aligned} \rho_{e} \biggl(\frac{\partial^{3}\eta_{1}}{\partial T^{3}} - V_{Ae}^{2} \frac{\partial^{3}\eta_{1}}{\partial T\partial Z^{2}} \biggr) &= \frac{1}{R}\frac{\partial\widetilde{P}_{1}}{\partial T}\cos\phi. \end{aligned}$$

Adding these two equations and assuming that $\eta_{1}$ is a periodic function of $T$ with a zero average we obtain

28 $$ \frac{\partial^{2}\eta_{1}}{\partial T^{2}} - C_{k}^{2}\frac{\partial^{2}\eta_{1}}{\partial Z^{2}} = 0, \quad C_{k}^{2} = \frac{2B^{2}}{\mu_{0}(\rho_{i} + \rho_{e})}. $$

We take $\eta_{1}$ proportional to $\sin(\Omega T)$. It follows from Equations 18 and 19 that $\eta_{1} = 0$ at $Z = \pm R/2$. Then we obtain the Sturm–Liouville problem for $\eta_{1}$,

29 $$ \frac{\partial^{2}\eta_{1}}{\partial Z^{2}} + \frac{\Omega^{2}}{C_{k}^{2}}\eta_{1} = 0, \quad \eta_{1} = 0 \quad \mbox{at} \ Z = \pm R/2. $$

The solution to this problem corresponding to the fundamental mode is proportional to $\cos(\pi Z/R)$. In accordance with Equations 26 and 27 we also take $\eta_{1}$ proportional to $\cos\phi$. Then we obtain

30 $$ \eta_{1} = R\sin(\Omega T)\cos\phi\cos(\pi Z/R), \quad \Omega = \pi C_{k}/R , $$

where we arbitrarily chose the proportionality coefficient equal to $R$. Now it is straightforward to obtain

31 $$ \textstyle\begin{array}{c} P_{1} = \frac{1}{2} R^{2}\Omega^{2}(\rho_{i} - \rho_{e})(r/R)f(r)\sin(\Omega T) \cos\phi\cos(\pi Z/R), \\ v_{r1} = R\Omega f(r)\cos(\Omega T)\cos\phi\cos(\pi Z/R), \\ v_{\phi1} = R\Omega g(r)\cos(\Omega T)\sin\phi\cos(\pi Z/R), \\ b_{r1} = \pi B f(r)\sin(\Omega T)\cos\phi\sin(\pi Z/R), \\ b_{\phi1} = \pi B g(r)\sin(\Omega T)\sin\phi\sin(\pi Z/R), \end{array} $$

where the function $g(r)$ is defined by

32 $$ g(r) = \left \{\textstyle\begin{array}{l@{\quad}l} -1, & r < R, \\ (R/r)^{2}, & r > R. \end{array}\displaystyle \right . $$

### The Second-Order Approximation

Now we collect terms of the order of $a^{2}$ in Equations 7 – 11 and the boundary conditions Equations 13 and 14. This yields

33 $$\begin{aligned} \frac{\partial v_{r2}}{\partial T} + \frac{1}{\rho}\frac{\partial P_{2}}{\partial r} - \frac{B}{\mu_{0}\rho}\frac{\partial b_{r2}}{\partial Z} &= \frac{1}{\mu_{0}\rho} \biggl(b_{r1}\frac{\partial b_{r1}}{\partial r} + \frac{b_{\phi1}}{r}\frac{\partial b_{r1}}{\partial\phi} - \frac{b_{\phi1}^{2}}{r} \biggr) \\ &\quad {}- v_{r1}\frac{\partial v_{r1}}{\partial r} - \frac{v_{\phi1}}{r} \frac{\partial v_{r1}}{\partial\phi} + \frac{v_{\phi1}^{2}}{r} , \end{aligned}$$

34 $$\begin{aligned} \frac{\partial v_{\phi2}}{\partial T} + \frac{1}{\rho r}\frac{\partial P_{2}}{\partial\phi} - \frac{B}{\mu_{0}\rho}\frac{\partial b_{\phi2}}{\partial Z} &= \frac{1}{\mu_{0}\rho} \biggl(b_{r1} \frac{\partial b_{\phi1}}{\partial r} + \frac{b_{\phi1}}{r}\frac{\partial b_{\phi1}}{\partial\phi} + \frac{b_{r1}b_{\phi1}}{r} \biggr) \\ &\quad {}- v_{r1}\frac{\partial v_{\phi1}}{\partial r} - \frac{v_{\phi1}}{r} \frac{\partial v_{\phi1}}{\partial\phi} - \frac{v_{r1}v_{\phi1}}{r} , \end{aligned}$$

35 $$\begin{aligned} \frac{\partial b_{r2}}{\partial T} - B\frac{\partial v_{r2}}{\partial Z} &= \frac{1}{r} \frac{\partial}{\partial\phi}(v_{r1} b_{\phi1} - v_{\phi1} b_{r1}), \end{aligned}$$

36 $$\begin{aligned} \frac{\partial b_{\phi2}}{\partial T} - B\frac{\partial v_{\phi2}}{\partial Z} &= -\frac{\partial}{\partial r}(v_{r1} b_{\phi1} - v_{\phi1} b_{r1}), \end{aligned}$$

37 $$\begin{aligned} \frac{\partial(rv_{r2})}{\partial r} + \frac{\partial v_{\phi2}}{\partial\phi} &= 0, \end{aligned}$$

38 $$\begin{gathered} v_{r2} = v_{\phi2} = 0 \quad \mbox{at} \ Z = \pm R/2 , \end{gathered}$$

39 $$\begin{gathered} v_{r2} - \frac{\partial\eta_{2}}{\partial T} = \frac{v_{\phi1}}{r}\frac{\partial\eta_{1}}{\partial\phi} - \eta_{1}\frac{\partial v_{r1}}{\partial r} , \qquad \biggl[\!\!\biggl[P_{2} + \eta_{1}\frac{\partial P_{1}}{\partial r} \biggr]\!\!\biggr]= 0 \quad \mbox{at} \ r = R . \end{gathered}$$

To solve this system of equations and boundary conditions we need to impose either the initial or periodicity conditions with respect to time on the variables of the second-order approximation. There are five Equations 33 – 37 for five variables. However, only the first four of them contain temporal derivatives because we dropped the temporal derivative of $b_{z}$ in the $z$-component of the induction equation. This implies that we can impose only four conditions related to the time. In what follows we assume that $v_{r2}$, $v_{\phi2}$, $b_{r2}$, and $b_{\phi2}$ are periodic functions of time with zero averages, while we do not impose any conditions on $P_{2}$.

Now we use Equations 30 and 31 to calculate the right-hand sides of Equations 33 – 36 and the nonlinear terms in the boundary conditions in Equation 39. As a result we obtain

40 $$\begin{aligned} \frac{\partial v_{r2}}{\partial T} + \frac{1}{\rho}\frac{\partial P_{2}}{\partial r} - \frac{B}{\mu_{0}\rho}\frac{\partial b_{r2}}{\partial Z} &= \frac{\pi^{2} R^{2}[f(r) + g(r)]}{r^{3}} \bigl[C_{k}^{2}\cos^{2}(\Omega T) \cos^{2}(\pi Z/R) \\ &\quad {} - V_{A}^{2}\sin^{2}( \Omega T)\sin^{2}(\pi Z/R) \bigr], \end{aligned}$$

41 $$\begin{aligned} \frac{\partial v_{\phi2}}{\partial T} + \frac{1}{\rho r}\frac{\partial P_{2}}{\partial\phi} - \frac{B}{\mu_{0}\rho} \frac{\partial b_{\phi2}}{\partial Z} &= 0, \end{aligned}$$

42 $$\begin{gathered} \frac{\partial b_{r2}}{\partial T} = B\frac{\partial v_{r2}}{\partial Z}, \qquad \frac{\partial b_{\phi2}}{\partial T} = B \frac{\partial v_{\phi2}}{\partial Z}, \qquad \frac{\partial(rv_{r2})}{\partial r} + \frac{\partial v_{\phi2}}{\partial\phi} = 0, \end{gathered}$$

43 $$\begin{gathered} v_{r2} - \frac{\partial\eta_{2}}{\partial T} = \frac{R\Omega}{4} \bigl\{ f(r) + \bigl[f(r) + 2g(r)\bigr]\cos2\phi \bigr\} \sin(2\Omega T)\cos^{2}(\pi Z/R) \quad \mbox{at} \ r = R, \end{gathered}$$

44 $$\begin{gathered} P_{e2} - P_{i2} = R^{2}\Omega^{2}( \rho_{i} - \rho_{e})\sin^{2}(\Omega T) \cos^{2}\phi\cos^{2}(\pi Z/R) \quad \mbox{at} \ r = R. \end{gathered}$$

When deriving Equation 40 we took into account that $f(r) + g(r) = 0$ when $r < R$.

Using Equation 42 to eliminate $b_{r2}$ and $b_{\phi2}$ from Equations 40 and 41 we obtain

45 $$\begin{gathered} \begin{aligned}[b] \frac{\partial^{2} v_{r2}}{\partial T^{2}} - V_{A}^{2} \frac{\partial^{2} v_{r2}}{\partial Z^{2}} + \frac{1}{\rho}\frac{\partial^{2} P_{2}}{\partial r\partial T} &= - \frac{\pi^{2} R^{2}\Omega[f(r) + g(r)]}{r^{3}} \bigl[C_{k}^{2}\cos^{2}(\pi Z/R) \\ &\quad {} + V_{A}^{2}\sin^{2}(\pi Z/R) \bigr]\sin(2\Omega T), \end{aligned} \end{gathered}$$

46 $$\begin{gathered} \frac{\partial^{2} v_{\phi2}}{\partial T^{2}} - V_{A}^{2}\frac{\partial^{2} v_{\phi2}}{\partial Z^{2}} + \frac{1}{\rho r}\frac{\partial^{2} P_{2}}{\partial\phi\partial T} = 0. \end{gathered}$$

Using Equation 37 to eliminate $v_{r2}$ and $v_{\phi2}$ from Equations 45 and 46 we obtain the equation for $P_{2}$:

47 $$\begin{aligned} r\frac{\partial}{\partial r}r\frac{\partial^{3} P_{2}}{\partial r\partial T} + \frac{\partial^{3} P_{2}}{\partial\phi^{2}\partial T} & = \frac{4\pi^{2}\rho R^{2}\Omega[f(r) + g(r)]}{r^{2}} \bigl[C_{k}^{2}\cos^{2}(\pi Z/R) \\ &\quad {} + V_{A}^{2}\sin^{2}(\pi Z/R) \bigr]\sin(2\Omega T). \end{aligned}$$

Differentiating Equation 44 with respect to time yields

48 $$\begin{aligned} \frac{\partial P_{e2}}{\partial T} - \frac{\partial P_{i2}}{\partial T} =& \frac{1}{2} R^{2}\Omega^{3}(\rho_{i} - \rho_{e}) \sin(2\Omega T) (1 + \cos2\phi)\cos^{2}(\pi Z/R) \quad \mbox{at} \ r = R. \end{aligned}$$

We look for the solution to Equation 47 satisfying the boundary condition of Equation 48 in the form

49 $$ \frac{\partial P_{2}}{\partial T} = \Omega \bigl[\widetilde{P}_{0}(r,Z) + \widetilde{P}_{2}(r,Z)\cos2\phi \bigr]\sin(2\Omega T). $$

Substituting this expression in Equations 47 and 48 we obtain the equations and boundary conditions

50 $$\begin{aligned} \frac{\partial}{\partial r}r\frac{\partial\widetilde{P}_{0}}{\partial r} &= \frac{4\pi^{2}\rho R^{2}[f(r) + g(r)]}{r^{3}} \bigl[C_{k}^{2}\cos^{2}(\pi Z/R) + V_{A}^{2}\sin^{2}(\pi Z/R) \bigr], \end{aligned}$$

51 $$\begin{aligned} \frac{\partial}{\partial r}r\frac{\partial\widetilde{P}_{2}}{\partial r} - \frac{4}{r} \widetilde{P}_{2} &= 0, \end{aligned}$$

52 $$\begin{aligned} \widetilde{P}_{e0} - \widetilde{P}_{i0} &= \widetilde{P}_{e2} - \widetilde{P}_{i2} = \frac{1}{2} R^{2}\Omega^{2}(\rho_{i} - \rho_{e}) \cos^{2}(\pi Z/R) \quad \mbox{at} \ r = R. \end{aligned}$$

The solutions to Equations 50 and 51 that are regular at $r = 0$, vanishing as $r \to \infty$, and satisfy the boundary conditions in Equation 52 are given by

53 $$\begin{aligned} \widetilde{P}_{0} =& \frac{\pi^{2}}{4} f(r) \bigl\{ C_{k}^{2}\bigl[(\rho_{i} - \rho_{e})g(r) - (\rho_{i} - 3\rho_{e})f(r)\bigr]\cos^{2}(\pi Z/R) \\ &{}+ 2\rho V_{A}^{2} f(r)\sin^{2}(\pi Z/R) \bigr\} , \end{aligned}$$

54 $$\begin{aligned} \widetilde{P}_{2} =& \frac{r^{2}}{4} f(r) \bigl[ \Omega^{2}(\rho_{i} - \rho_{e}) g(r) \cos^{2}(\pi Z/R) + f(r)Q_{2}(Z) \bigr], \end{aligned}$$

where $Q_{2}(Z)$ is an arbitrary function. Now we use Equations 43, 49, 53, and 54 to write Equation 45 as $r \to R$ from inside and outside. As a result we obtain

55 $$\begin{aligned} &\rho_{i}\frac{\partial}{\partial T} \biggl(\frac{\partial^{2}\eta_{2}}{\partial T^{2}} - V_{Ai}^{2}\frac{\partial^{2}\eta_{2}}{\partial Z^{2}} \biggr) + \frac{1}{2} R \Omega Q_{2}(Z)\sin(2\Omega T)\cos2\phi \\ &\quad = \frac{\pi^{2}\Omega C_{k}^{2}}{2R}\sin(2\Omega T) \bigl[\rho_{i} + ( \rho_{i} - \rho_{e})\cos2\phi\cos^{2}(\pi Z/R) \bigr] , \end{aligned}$$

56 $$\begin{aligned} &\rho_{e}\frac{\partial}{\partial T} \biggl(\frac{\partial^{2}\eta_{2}}{\partial T^{2}} - V_{Ae}^{2}\frac{\partial^{2}\eta_{2}}{\partial Z^{2}} \biggr) - \frac{1}{2} R \Omega Q_{2}(Z)\sin(2\Omega T)\cos2\phi \\ &\quad = \frac{\pi^{2}\Omega C_{k}^{2}}{2R}\sin(2\Omega T) \bigl[\rho_{e} + ( \rho_{i} - \rho_{e})\cos2\phi\cos^{2}(\pi Z/R) \bigr] . \end{aligned}$$

Now we impose the condition that $\eta_{2}$ is a periodic function of time with the zero average. Then, adding these two equations and integrating the obtained equation with respect to $T$ yields

57 $$ \frac{\partial^{2}\eta_{2}}{\partial T^{2}} - C_{k}^{2}\frac{\partial^{2}\eta_{2}}{\partial Z^{2}} = - \frac{\pi^{2} C_{k}^{2}}{4R}\cos(2\Omega T) \biggl(1 + \frac{\rho_{i} - \rho_{e}}{\rho_{i} + \rho_{e}}\cos2\phi \cos^{2}(\pi Z/R) \biggr). $$

We look for the solution to this equation in the form $\eta_{2} = \tilde{\eta}_{2}\cos(2\Omega T)$. As a result we obtain

58 $$ \frac{\partial^{2}\tilde{\eta}_{2}}{\partial Z^{2}} + \frac{4\pi^{2}\tilde{\eta}_{2}}{R^{2}} = \frac{\pi^{2}}{8R} \biggl(2 + \frac{\rho_{i} - \rho_{e}}{\rho_{i} + \rho_{e}} \cos2\phi\bigl[1 + \cos(2\pi Z/R)\bigr] \biggr). $$

In follows from Equations 38 and 43 that $\tilde{\eta}_{2}$ must satisfy the boundary conditions

59 $$ \tilde{\eta}_{2} = 0 \quad \mbox{at}\ Z = \pm R/2. $$

The general solution to Equation 58 with the boundary conditions in Equation 59 contains the term $C\sin(2\pi Z)$, where $C$ is an arbitrary constant. To eliminate this term we impose a viable condition that the solution must be symmetric with respect to the $Z = 0$ plane. This condition implies that $C = 0$. Then the solution is

60 $$ \tilde{\eta}_{2} = \frac{R}{32} \biggl[ \biggl(2 + \frac{\rho_{i} - \rho_{e}}{\rho_{i} + \rho_{e}} \cos2\phi \biggr) \biggl(1 + \cos\frac{2\pi Z}{R} \biggr) + \frac{\rho_{i} - \rho_{e}}{ \rho_{i} + \rho_{e}}\frac{\pi Z}{R}\cos2\phi\cos\frac{2\pi Z}{R} \biggr]. $$

Now, recalling the definition of $\tilde{\eta}_{2}$ and returning to the initial non-scaled variables, we eventually obtain

61 $$ \eta_{2} = \frac{R}{16}\cos(2\omega t) \biggl[2 \cos^{2}\frac{\pi z}{L} + \frac{\rho_{i} - \rho_{e}}{\rho_{i} + \rho_{e}}\cos2\phi \biggl( \cos^{2}\frac{\pi z}{L} + \frac{\pi z}{2L}\sin\frac{2\pi z}{L} \biggr) \biggr]. $$

We see that $\eta_{2}$ oscillates with a frequency equal to the double frequency of the kink oscillation. The second-order boundary perturbation is a superposition of the sausage perturbation that is independent of $\phi$ and the fluting perturbation that is proportional to $\cos2\phi$. It is easy to show that the amplitude of the fluting perturbation takes its maximum at $z = 0$ and monotonically decreasing when $|z|$ increases from 0 to $L/2$. This result is in complete agreement with the numerical results reported by Terradas, Magyar, and Van Doorsselaere (2017).

## Summary and Conclusions

In this article we studied the excitation of a fluting perturbation of the boundary of a magnetic flux tube by an imposed kink oscillation. We used the MHD equations in the approximation of cold plasmas, *i.e.* we neglected the plasma pressure in comparison with the magnetic pressure. We also used the thin-tube approximation and scaled the dependent and independent variables accordingly. Using this approximation enables us to eliminate the velocity component parallel to the background magnetic field and reduce the axial component of the induction equation to the condition that the plasma motion is incompressible.

To study the excitation of fluting perturbations by a kink oscillation we used the regular perturbation method where the dimensionless amplitude of the kink oscillation is used as a small parameter. The solution of the first-order approximation describes the kink oscillation. To solve the equations of the second-order approximation we imposed the periodicity conditions with respect to time on the dependent variables. The axial component of the induction equation contains the time derivative of the axial component of the magnetic-field perturbation. However, as we have already pointed out, in the thin-tube approximation this equation reduces to the condition that the plasma motion is incompressible and, thus, the time derivative is eliminated. As a result, the order of the system of MHD equations with respect to time reduces from five to four. This implies that we can impose only four periodicity conditions with respect to time. Hence, we imposed the conditions that the radial and azimuthal components of the velocity and magnetic-field perturbation are periodic functions of time with the zero averages, while we did not impose any conditions on the magnetic pressure perturbation.

We solved the equations of the second-order approximation and obtained the expression for the tube boundary perturbation. This perturbation is a superposition of sausage and fluting perturbation. Both perturbations oscillate with the frequency equal to the double frequency of the kink mode. The amplitude of the fluting perturbation takes its maximum at the middle of the tube and monotonically decreases with the distance from the middle point. The first overtone with respect to the axial variables of the first fluting mode is not excited. These results are in a complete agreement with the numerical results obtained by Terradas, Magyar, and Van Doorsselaere (2017).
